# Enhanced Viability of Endothelial Colony Forming Cells in Fibrin Microbeads for Sensor Vascularization 

**DOI:** 10.3390/s150923886

**Published:** 2015-09-18

**Authors:** Jarel K. Gandhi, Lada Zivkovic, John P. Fisher, Mervin C. Yoder, Eric M. Brey

**Affiliations:** 1 Department of Biomedical Engineering, Wishnick Hall 223, 3255 South Dearborn Street, Chicago, IL 60616, USA; E-Mail: jgandhi2@hawk.iit.edu; 2 Department of Physiology, Faculty of Pharmacy, University of Belgrade, Belgrade 11000, Serbia; E-Mail: lada@pharmacy.bg.ac.rs; 3 Fischell Department of Bioengineering, University of Maryland, College Park, MD 20742, USA; E-Mail: jpfisher@umd.edu; 4 Wells Center for Pediatric Research, Indiana University School of Medicine, Indianapolis, IN 46201, USA; E-Mail: myoder@iupui.edu; 5 Research Service, Hines Veterans Administration Hospital, Hines, IL 60141, USA

**Keywords:** tissue engineering, vascularization, bioreactor, long-term sensors, implantable sensors, fibrin

## Abstract

Enhanced vascularization at sensor interfaces can improve long-term function. Fibrin, a natural polymer, has shown promise as a biomaterial for sensor coating due to its ability to sustain endothelial cell growth and promote local vascularization. However, the culture of cells, particularly endothelial cells (EC), within 3D scaffolds for more than a few days is challenging due to rapid loss of EC viability. In this manuscript, a robust method for developing fibrin microbead scaffolds for long-term culture of encapsulated ECs is described. Fibrin microbeads are formed using sodium alginate as a structural template. The size, swelling and structural properties of the microbeads were varied with needle gauge and composition and concentration of the pre-gel solution. Endothelial colony-forming cells (ECFCs) were suspended in the fibrin beads and cultured within a perfusion bioreactor system. The perfusion bioreactor enhanced ECFCs viability and genome stability in fibrin beads relative to static culture. Perfusion bioreactors enable 3D culture of ECs within fibrin beads for potential application as a sensor coating.

## 1. Introduction

Many applications in tissue engineering and regenerative medicine require the ability to regulate the process of vessel assembly. Seeding of endothelial cells (ECs) in biomaterials *in vitro* prior to implantation is a popular option for enhancing *in vivo* vascularization post implantation. These EC containing materials can then be used as coating to enhance sensor function post-implantation. Vascularization locally at the sensor interface allows for a reduced diffusional distance, enabling more immediate and accurate readings [1,2]. It also has the potential to reduce the inflammatory and fibrosis responses to the sensor [3]. One potential application includes pre-vascularization of glucose sensors to improve long term function in type 1 diabetics. The general aim of EC seeding has been to utilize biomaterials scaffolds to provide a surface for cells to bind to improve viability and function, as well as provide a protective barrier from mechanical forces [4]. Fibrin, a naturally occurring polymer hydrogel, is a highly-suitable scaffold for EC seeding and sensor encapsulation. Fibrin displays good EC adhesion, sustained EC viability, and pro-vascularization properties [5,6,7]. Previous use of VEGF-releasing fibrin gels to induce neovascularization around a glucose sensor has resulted in an increase of vessel density, a reduction in fibrosis and an increase in sensor output [3]. The addition of ECs to this fibrin coating may further accelerate the rate of functional vessel formation.

One population of ECs that has been proposed for vascularization applications is endothelial colony-forming cells (ECFC). ECFCs are viable circulating cells that show clonal proliferative potential, with cobblestone appearance in monolayer cultures, and *in vivo* formation of human blood vessels upon implantation within a scaffold, when differentiated from umbilical cord blood or adult peripheral blood [8]. ECFCs are derived from circulating blood cells by culturing on collagen-coated plates with differentiation media [9,10]. The significant advantage of ECFCs is that they can easily be isolated from adult peripheral blood, enabling auto-transplantation. Further, ECFC delivery has been characterized as suitable for application to a wide variety of tissues, including bone [11,12], kidney [13,14], and neural tissue [15,16,17,18].

ECFCs have shown affinity for natural polymer-based scaffolding, including fibrin [19], rat collagen [20,21], porcine collagen [22] and Matrigel [19]. In general, within these biomaterials, ECFCs have shown the ability to form tubule-like structures *in vitro* and capillary vessels *in vivo*. Implantation of ECFC-seeded scaffolds has previously been utilized for *in vivo* tissue regeneration in various rodent models. ECFCs, seeded in collagen, have shown formation of vessel-like structured after subcutaneous implantation in severe combined immunodeficiency (SCID) mice [22]. Additionally, ECFCs, in co-culture with mesenchymal progenitor cells (MPCs), have been utilized with Matrigel plugs to form vascularized scaffolds once implanted subcutaneously in SCID mice [23]. The formed vessel-structures showed anastomosis between the host and the scaffold via tail-vein perfusion of fluorescent lectin molecules. These studies suggest the substantial potential of ECFCs for tissue engineering application. However, the scaffold volumes involved in previous studies would be difficult to translate into clinical application.

One proposed solution is the combination of fibrin microbead scaffolds with a perfusion bioreactor system. Fibrin microbeads have previously been generated using an oil emulsion for delivery of mesenchymal stem cells (MSCs) [24]. However, this procedure requires multiple washes with organic chemicals. The use of organic chemicals may affect cell viability during formation, and residual amounts within the scaffold could induce inflammatory responses *in vivo*. The formation of fibrin bead scaffolds for *in vitro* chondrocyte tissue engineering has also previously been demonstrated [25]. This technique utilizes alginate as a template for the fibrin to polymerize into a spherical shape. The hybrid bead scaffolds have been shown to improve cryopreservation of bone marrow-derived stem cells (BMSCs) [26]. This approach holds promise for EC culture but has not been optimized for this application.

A tubular perfusion bioreactor system (TPS) has previously been shown to enable culture of MSCs within 3D alginate spherical scaffolds [27]. The TPS consists of a tubular growth chamber that, when packed with spherical scaffolds, void spaces between the scaffolds enhance convective transport through the growth chamber. This enables long-term culture of multiple scaffolds within the growth chamber, providing the potential for growth of clinically-relevant volumes. Using poly (lactic-co-glycolic acid)/poly(ε-caprolactone) scaffolds, the TPS system has shown successful bone regeneration following delivery of osteogenic-differentiating MSCs in a rat femoral condyle defect [28].

In this study, we describe a method for generating fibrin microbeads suitable for scalable, 3D ECFC culture in a perfusion bioreactor system. We describe the influence of synthesis conditions on the resulting fibrin microsphere properties. The use of the fibrin bead scaffolds for scaffolding of ECs is demonstrated with ECFCs and Human umbilical vein endothelial cells (HUVECs), and we have tested the influence of these conditions on cell viability and DNA damage induction for ECFCs in the various culture environments. We report that the TPS system enhances ECFC viability and reduces DNA damage in 3D scaffolds and may hold promise in vascularizing sensors for improved long-term functionality [27].

## 2. Experimental Section

### 2.1. Cell Culture

Human umbilical vein endothelial cells (HUVECs) were purchased from Lonza (Gaithersburg, MD, USA). HUVECS were used from passage 3–8. Endothelial colony forming cells (ECFCs) were a generous gift from Dr. Mervin C. Yoder, Indiana University School of Medicine. The ECFCs used were obtained from human umbilical cord blood as previously described [29]. ECFCs were used from passage 2–4, prior to clustering phenotype dissipation. Complete Endothelial Growth Media (EGM-2) (Lonza) was utilized for both cell types.

### 2.2. Fibrin Bead Formation

**Figure 1:**
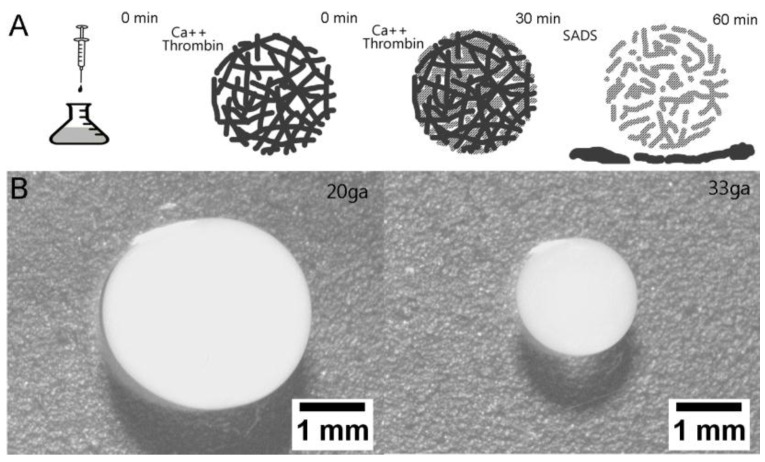
Fibrin Beads Formation: (**A**) Schematic showing fibrin bead formation. Alginate (black) will immediately cross-linking due to the presence of Ca^2+^. Over time, thrombin in the cross-link solution will diffuse into the bead, cleaving fibrinogen to fibrin (grey), allowing for self-polymerization. These beads are then incubated in SADS to chelate the Ca^2+^, dissolving the alginate and leaving a fibrin bead scaffold; (**B**) Representative bright-field images of fibrin beads formed with 20 ga and 33 ga needle.

Fibrin beads were prepared using a method based on a sacrificial alginate template (Figure 1A). For the precursor solution, 1.2% w/w sodium alginate low viscosity, high guluronate content (LVG) (Pronova, Sandvika, Norway) was mixed with various concentrations (1, 2, 3, 5, 10, 15, 25, 40, 80 mg/mL) of fibrinogen (Sigma-Aldrich, St Louis, MO, USA) in a solution consisting of 50 mM NaCl, 10 mM KCl, 250 mM HEPES, 100 ug/mL glucose, and 0.02% EDTA. The precursor solution was carefully mixed, with or without cells (2 million cells/mL). The precursor solution was loaded into a 1mL syringe (BD; Franklin Lakes, NJ, USA), and a blunt tip needle (20, 26, 30, 33 ga) (Hamilton, Reno, NV, USA) was attached. The precursor solution was added, dropwise, to a cross-linking solution of 1.1% CaCl_2_, and 40 U/mL human thrombin (Red Cross, Washington, DC, USA). Beads were incubated in the cross-linking solution for 30 min, removed and then washed twice with Phosphate-Buffered Saline (PBS). The alginate was extracted from the beads using a sodium alginate dissolution solution (SADS), consisting of 50 mM NaCl, 150 mM Sodium Citrate, and 30 mM EDTA in TBS buffer, pH 7.4. The beads were incubated in the dissolution solution for 20 min at 37 °C. Beads were washed twice in PBS and incubated in EGM-2 media ±100 U/mL aprotinin (Sigma-Aldrich, St. Louis, MO, USA).

### 2.3. Swelling Ratio and Imaging

The fibrin beads were incubated in dH_2_O for 2 h to allow complete swelling. Beads were then imaged under a surgical microscope (Fisher Science, Pittsburgh, PA, USA), with an attached 1.4″ CCD, 0.3 MP camera (Imaging Source, Bremen, Germany). Images were analyzed for size using a custom MATLAB (Mathworks, Natick, MA, USA) code. Twenty beads were removed from the solution using a spatula, dried briefly with a kimwipe, and weighed to determine the wet mass (M_wet_). Beads were then dried in a vacuum oven at 37 °C overnight. A dry mass (M_dry_) was then obtained. The swelling ratio was calculated as (M_wet_-M_dry_)/M_dry_. Five batches were analyzed per condition.

### 2.4. Histology

Beads with and without cells incubated for 0, 1, 2, and 7 days were fixed for 10 min in 10% Formalin (Fisher Science, Waltham, MA, USA). Beads were then washed with PBS, paraffin embedded and sectioned (30 µm thickness) for histological staining. Sample sections were deparaffinized and washed with PBS. Sections were stained either with 1x Coomassie Blue (Sigma-Aldrich) for 1 h, or 100 mg/mL Hoechst 33,258 stain (Life Technologies, Carlsbad, CA, USA) for 5 min. Slides were again washed, and mounted with Aqua mount (Fisher Science).

### 2.5. Scanning Electron Microscopy

Ten beads from 3 batches were fixed with 2.5% glutaraldehyde in PBS for 10 min, washed with PBS, flash frozen using liquid nitrogen, and lyophilized overnight. Beads were then glued to a scanning electron microscope (SEM) mount, sputter coated (Polaron, Laughton, UK) with gold, and imaged using SEM (JEOL; Tokyo, Japan), at the Research Resources Center electron microscopy core at the University of Illinois-Chicago.

### 2.6. Live/Dead Assay

Five beads with cells were incubated in EGM-2 for 24 h in static culture after formation. They were then stained using a Live/Dead Assay (Life Technologies) for 30 min, following the manufacturer’s protocol. The samples were imaged with a 5× objective using confocal microscopy (Leica, Buffalo Grove, IL, USA), using PASCAL software (Leica). Excitation was applied using 488 nm and 543 nm lasers, and images were obtained using 505 nm and 560 nm long pass filters, respectively. Serial images were taken and reconstructed using PASCAL software. Four batches were analyzed per condition.

### 2.7. Viability Assay

Slides stained with Hoechst 33,258 were imaged with fluorescence microscopy (Leica) with a 5× objective, using 350 nm excitation and 458 nm emission filter. Multiple images were taken and digitally stitched into a mosaic using Axiovision. Randomly chosen beads for each condition were analyzed, with all quantitative values averaged. Three batches were analyzed per condition.

Images of Hoechst stained slides were analyzed using Image J software (NIH, Bethesda, MD, USA). Nuclei were quantified using the cell counter tool. The total cross-sectional area of the microsphere was calculated by manual tracing. Cell density values were calculated as the total nuclei count divided by the cross-sectional area. Cell densities were normalized by dividing by day 0 values and presented as percentages.

### 2.8. Bioreactor Culture

A closed-circuit tubular perfusion bioreactor system (TPS) was utilized to culture ECFCs embedded in fibrin bead scaffolds. The TPS was setup as previous described, with modifications [27,28,30,31,32,33,34]. An L/S Multichannel Pump System (Cole Parmer, Vernon Hills, IL, USA) was utilized at a 3 mL/min flow rate. Sterile tubing was assembled in an incubator with platinum-cured silicone tubing (Cole Parmer). The growth chamber consisted of an approximately 13 cm-long platinum-cured silicone tube (Cole Parmer) with an inner diameter of 6.4 mm. The chamber was loaded with 10 ECFC-embedded beads loaded via transfer pipette. A 200 mesh stainless steel screen was added to prevent mobilization of the beads. EGM-2 media was added to a 100 mL glass bottle reservoir that was connected to the tubing. Media change was performed every 2 days, by briefly pausing the flow, aspirating the media, and adding fresh media aseptically via syringe. Beads were gathered for analysis after 0, 2, and 7 days by removal from the growth chamber, and washing with PBS. Bioreactor cultured beads were processed and analyzed as described above.

### 2.9. Comet Assay to Assess DNA Damage

The comet assay was performed as previously described, with modification [35]. ECFCs were cultured on tissue-culture polystyrene (2D) or embedded in 26 ga, 40 mg/mL fibrinogen fibrin beads and cultured within the TPS with a flow rate of 3 mL/min, or statically in a petri dish. All conditions were cultured for 7 days, with media change every 2 days. Cells were isolated from the fibrin beads using 0.05% Trypsin/EDTA for up to 30 min, to completely dissolve the fibrin gels. Cells were centrifuged at 400 g for 5 min. 10,000 cells in 10 uL of media were embedded in 1% low melting-point (LMP) agarose (Sigma-Aldrich) in PBS, and layered on top of a glass microscope slide with pre-coated 1% normal-melting point agarose, and incubated at 4 °C for 5 min to fully gel. Once hardened, a third layer of 0.67% LMP agarose was added and incubated at 4 °C for 5 min.

Slides were placed in cold lysing solution, pH 10, containing 2.5 M NaCl, 100 mM EDTA, 10 mM Tris, 1% Triton X-100 and 10% dimethylsulfoxide and incubated at 4 °C overnight. Slides were then subjected to electrophoresis for 30 min at 25 V (300 mA). Slides were neutralized in 0.4 M Tris, pH 7.5 buffer for 15 min, washed, and stained with 20 µg/L ethidium bromide. The comets were imaged under fluorescent microscope, 40x objective, using 350 nm excitation and 458 nm emission filter. Evaluation of DNA damage was done, as previously described, with modification [36]. DNA damage in the cells was assessed by quantification of the amount of DNA released from the core of the nucleus. Comets were visually scored and classified into three categories corresponding to the extent of DNA migration: (1) no damage, <5%, (2) moderate levels of damage, 5%–40%, and (3) severe levels of damage, 40%–100%. Analysis was performed on 100 randomly selected cells per sample.

### 2.10. Statistics

All statistical calculations were performed using Excel software (Microsoft, Redlands, WA, USA) and SigmaPlot software (Systat Software, San Jose, CA, USA). Regression tests were utilized to verify trends on bead formation parameters and an F test was utilized to determine statistical significance. Student’s t tests were used to test statistical significance of the Live/Dead assay results. A two-way ANOVA test, using the Holm-Sidak method, was used to test significance of time-dependent nuclei count. A one-way ANOVA test, using the tukeys method, was used to test significance of cell DNA damage. Statistical probability values *p* < 0.05 were considered significant and are denoted with an asterisk.

## 3. Results

### 3.1. Formation and Control of Fibrin Beads

Fibrin does not easily assemble into spherical structures using standard crosslinking methods due to loss of shape during the time required for thrombin-induced polymerization of fibrinogen. Using alginate as a structural template, fibrinogen could be assembled into spherical scaffolds that maintained their structure during exposure to thrombin. The alginate was then removed by incubation in SADS leaving a fibrin microsphere (Figure 1A). Following the procedure, fibrin beads appeared opaque, firm, and spherical (Figure 1B). The surface was smooth, and the beads did not stick to polystyrene surfaces. Bead formation occurred with fibrinogen concentrations of 1 mg/mL to 80 mg/mL. Beads formed with fibrinogen concentrations above 3 mg/mL retained their shape and did not collapse when removed from supporting liquid. At concentrations below 3 mg/mL a portion of the beads would deform during handling. At very high concentrations (80 mg/mL), beads tended to stick to one another.

**Figure 2:**
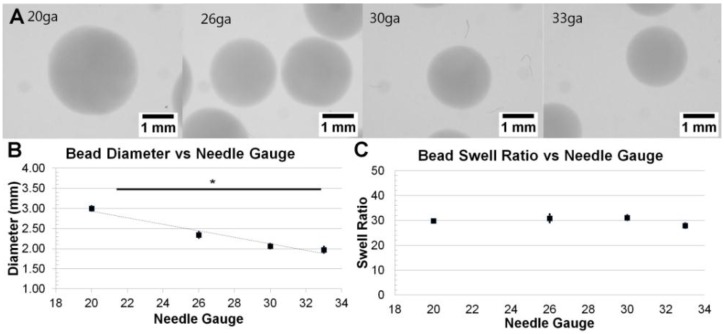
Variation of Bead Diameter with Needle Gauge: (**A**) Representative images of fibrin beads at various gauge needles; (**B**) Graph depicting the dependence of bead diameter on needle gauge (*p* < 0.05); (**C**) Graph showing no general relationship between needle gauge and bead swell ratio (*p* > 0.05).

While holding fibrinogen concentrations constant at 40 mg/mL (the median of the varied range), the needle gauge was varied between 20 ga and 33 ga in order to investigate its effect on bead size. Qualitatively, beads were observed to vary in size but not opacity (Figure 2A). Quantitative analysis showed that beads formed with a 20 ga needle were 3.00 ± 0.06 mm in diameter, while 26 ga were 2.34 ± 0.09 mm, 30 ga were 2.06 ± 0.06 mm, and 33 ga were 1.97 ± 0.10 mm (Figure 2B). These results show that the bead diameter significantly varied inversely with needle gauge (*p* = 0.0218). Swelling ratios values were: 29.82 ± 1.20 for 20 ga, 30.82 ± 3.68 for 26 ga, 31.12 ± 2.28 for 30 ga, and 27.92 ± 2.19 for 33 ga (Figure 2C). These data do not suggest any significant correlation between needle gauge and swell ratio when using a constant fibrinogen concentration (*p* = 0.6461).

**Figure 3:**
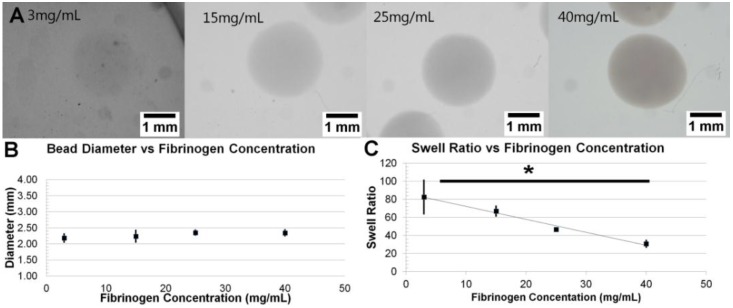
Variation of Bead Swelling Ratio with Fibrinogen Concentration: (**A**) Representative images of fibrin beads at various fibrinogen concentrations; (**B**) Graph showing no general relationship between bead diameter and fibrinogen concentrations (*p* > 0.05); (**C**) Graph depicting the dependence of bead swell ratio on fibrinogen concentration (*p* < 0.05).

While holding needle gauge constant at 26 ga (the median of the varied range), the fibrinogen concentration in the precursor solution was varied between 3 mg/mL and 40 mg/mL. Beads formed with fibrinogen concentrations less than 3 mg/mL were difficult to handle and often collapsed. Qualitatively, beads varied in opacity, with opacity increasing with fibrinogen concentration; however, size did not vary (Figure 3A). Beads formed with fibrinogen concentrations of 3, 15, 25 and 40 mg/mL had diameters of 2.18 mm ± 0.12 mm, 2.24 ± 0.18 mm, 2.35 ± 0.07 mm and 2.34 ± 0.09 mm, respectively (Figure 3B). There was no statistically significant correlation between fibrinogen concentration and size (*p* = 0.0982). Swelling ratios values include: 82.54 ± 18.47 for 3 mg/mL, 66.86 ± 5.40 for 15 mg/mL, 46.63 ± 1.92 for 25 mg/mL, and 30.81 ± 3.68 for 40 mg/mL (Figure 3C). A statistically significant inverse correlation is seen between initial fibrinogen concentration and swelling ratio (*p* = 0.0074). Swelling ratio often correlates with gel stiffness. These results suggest that higher fibrinogen concentration results in increasing gel stiffness.

### 3.2. Fibril Formation Analysis of Fibrin Beads

**Figure 4:**
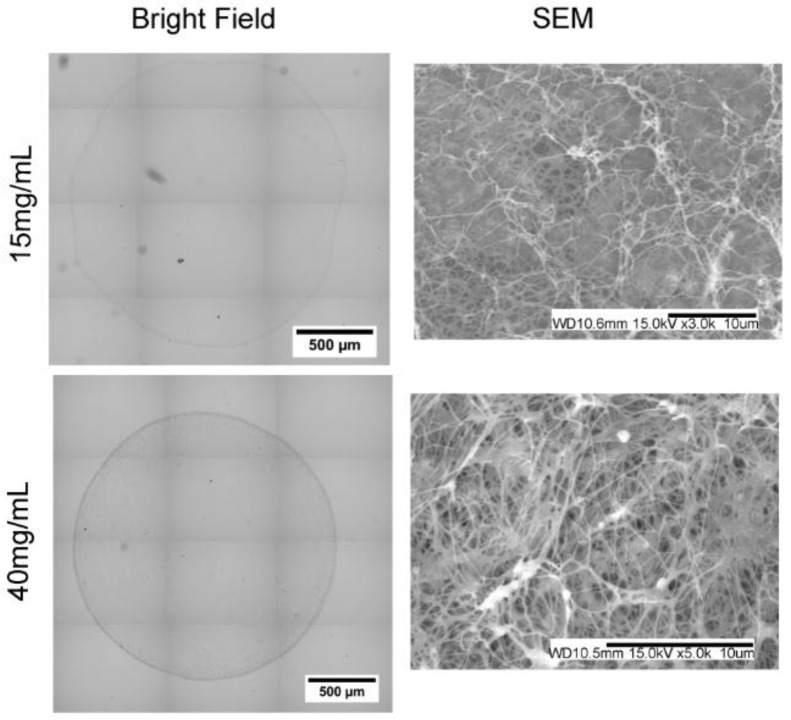
Bead Cross-Section and SEM.

Fibrin beads without cells were first processed for histology and stained with coomassie blue to visualize protein distribution (Figure 4). Overall, the stains revealed solid microbead structures with uniform staining intensity. The use of low purity alginate or lower thrombin concentrations resulted in microspheres with hollow cores or non-uniform staining (data not shown). Fibrin beads were processed and imaged with SEM to evaluate fibril structure. SEM images show fiber structures with sizes similar to previous literature for bulk fibrin gels [37]. The fibers density was higher in samples with higher fibrinogen concentrations.

### 3.3. Stability of Fibrin Beads in Culture

**Figure 5:**
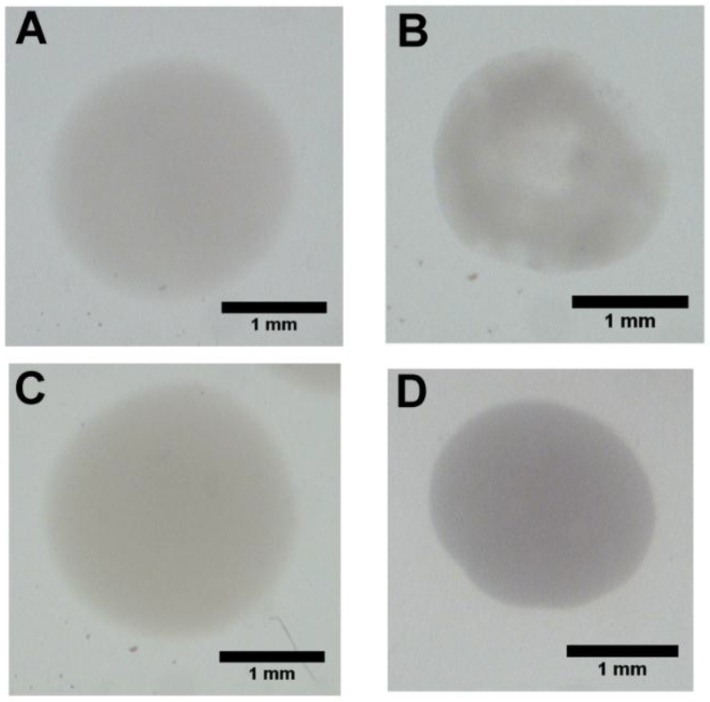
Longevity of Bead Structure: Beads were incubated in EGM-2 media and imaged at day 0 (**A**), day 4 (**B**), and day 5 (**C**). Beads incubated in media supplemented with 100 U/mL Aprotinin were imaged at 1 month (**D**).

Fibrin beads were first cultured statically. Bead failure was assessed qualitatively as the loss of spherical shape and increase in particulate matter. Compared to day 0 samples (Figure 5A), fibrin beads cultured in EGM-2 appear to show signs of degradation, as evidenced by variations in opacity, by day 4 and were completely degraded with no bulk scaffold remaining by day 5 (Figure 5B,C). This observation is independent of fibrinogen concentration or the presence of cells. To test if fibrin bead stability could be prolonged, 100 U/mL aprotinin was added to the media throughout culture. Fibrin beads cultured in aprotinin-supplemented media remained stable out to our longest time point of 1 month without any visible signs of degradation or particulate matter (Figure 5D). No side effects were noticeable through the inclusion of aprotinin.

### 3.4. 24 h Cell Viability

**Figure 6:**
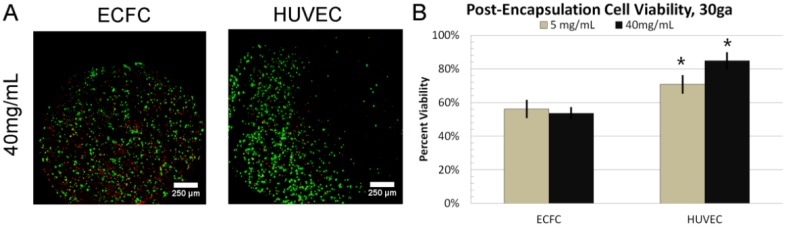
Cell Viability 24h Post-Encapsulation: (**A**) Image showing live/dead staining assay for various types of endothelial cells; (**B**) Graph showing viability data for ECFCs and HUVECs, at 5 or 40 mg/mL fibrinogen concentration (* indicates *p* < 0.05).

To test cell survival through the process of bead formation, ECFCs, at a concentration of 2 × 10^6^ cells/mL, were suspended in the precursor solution prior to gelation. All beads were formed using a 30 ga needle, a small diameter needle, with 5 or 40 mg/mL fibrinogen concentration, the low and high values of the formation range. After formation, the beads were cultured statically for 24 h with aprotinin-supplemented media. Qualitatively, cells are seen distributed uniformly throughout the scaffold, with both live and dead cells randomly located (Figure 6A). Quantitatively, ECFC viability was 54% ± 4% for 40 mg/mL, and 56% ± 5% for 5 mg/mL fibrinogen (*p* = 0.5297) (Figure 6B). The same experiment was performed with HUVECS. The distribution and location of live cells and dead cells appeared similar to ECFCs. However, HUVEC viability was 85% ± 6% for 40 mg/mL, and 71% ± 4% for 5 mg/mL fibrinogen concentration beads (*p* = 0.0355). Compared to ECFC viability, HUVEC viability was significantly different for 40 mg/mL (*p* = 0.0023) and 5 mg/mL (*p* = 0.0189).

### 3.5. Dynamic Culture 

**Figure 7:**
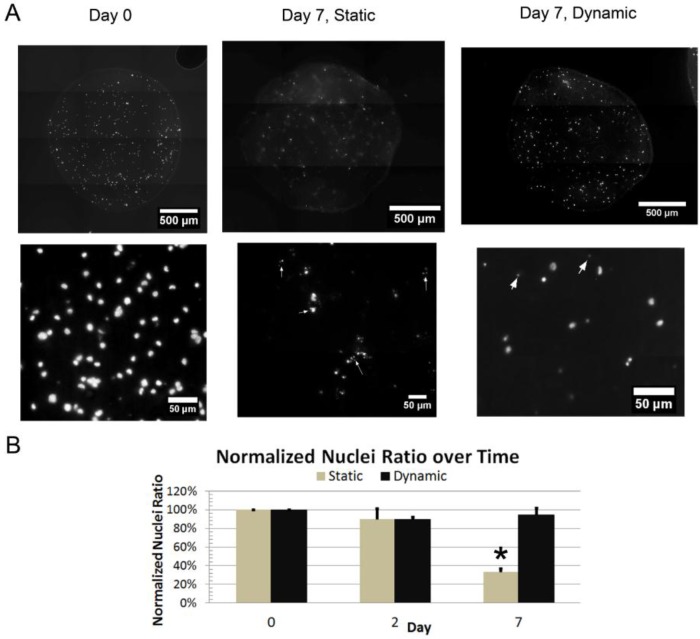
Nuclear Staining of Fibrin Beads with ECFCs: (**A**) Images depicting fibrin bead cross-sections stained for nuclei at day 0 and day 7 for beads cultured statically or dynamically; (**B**) Graph showing the normalized nuclei count up to day 7 (* indicates *p* < 0.05).

ECFCs suspended in fibrin beads formed with a fixed fibrinogen concentration of 40 mg/mL were cultured in both static conditions and using a perfusion bioreactor system (TPS). A 3 mL/min flow rate was utilized within the TPS, which was the lowest flow rate previously shown to provide transport benefit [27]. Beads were processed for histology, and cross-sections were stained for nuclei. At Day 0, ECFC nuclei are seen uniformly distributed throughout the bead scaffold cross-section (Figure 7A). The average nuclei count per mm^2^ at day 0 was 147 ± 76 (defined as 100%). In static culture, at day 2, the normalized nuclei count ratio was 89% ± 45%, and at day 7 was 33% ± 15% (Figure 7B). Over this time period, there is an increase in the amount of punctate, apoptotic structures found in the nuclear stains (arrows in Figure 7A).

When cultured within the TPS at a media flow rate of 3mL/min, the normalized nuclei count ratio was 94% ± 1% at day 2, and 94% ± 28% at day 7 (Figure 7B). Qualitatively, we see that the majority of nuclei stained are large and diffuse, and that the nuclei are scattered throughout the cross-section of the bead. Comparing the dynamic and static culture normalized nuclei ratios, there was a decrease over time in the statically cultured samples but not in the dynamic cultured samples. The statically-cultured day 7 sample is significantly different from the static day 0 (*p* = 0.001), day 7 (*p* = 0.003) and the dynamic day 7 sample (*p* = 0.002). This shows that the perfusion bioreactor increased ECFC viability when suspended in 3D fibrin bead scaffolds.

### 3.6. DNA Damage

**Figure 8:**
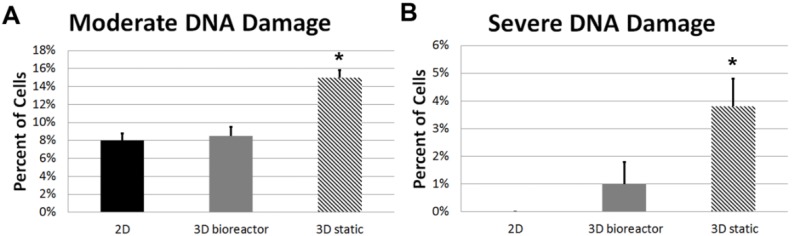
Comet Assay for DNA Damage: Graphs showing the percent of cells with (**A**) moderate and (**B**) severe DNA damage (* indicates *p* < 0.05).

To quantify potential DNA damage, the comet assay was performed on 2D-cultured ECFCs, 3D TPS-cultured ECFCs, and 3D static-cultured ECFCS and results are expressed as percent of cells without damage, moderate damage and severe damage. ECFCs cultured 2D on tissue-culture polystyrene showed minimal levels of DNA damage, with 92.0% ± 0.8% of cells showing no damage, 8.0% ± 0.8% moderate damage, and 0% severe damage. ECFCs embedded in 3D fibrin beads (26 ga, 40 mg/mL fibrinogen) and cultured dynamically with a 3 mL/min flow for 7 days, showed similar levels of DNA damage, with 90.5% ± 1.3% of cells showing no damage, 8.5% ± 1.0% moderate damage, and 1.0% ± 0.8% severe damage. ECFCs embedded in the same fibrin beads, but cultured statically for 7 days showed increased levels of DNA damage, with 81.3% ± 1.5% of cells showing no damage, 15.0% ± 0.8% moderate damage, and 3.8% ± 1.0% severe damage. There was an overall reduction in cells isolated for analysis in the 3D static-cultured group compared to TPS-cultured group, as expected from earlier results. ECFCs cultured within fibrin beads under static condition showed statistically significant increase of moderate damage compared to 2D culture (*p* < 0.001) and 3D TPS-culture (*p* < 0.001), as well as of severe damage compared to 2D culture (*p* < 0.001) and 3D TPS-culture (*p* = 0.001) (Figure 8). There was no statistical difference between ECFCs cultured 2D or 3D in the TPS for moderate (*p* = 0.711) or severe DNA damage (*p* = 0.182). This shows that TPS-culture of ECFCs within fibrin beads reduces DNA damage compared to static culture.

## 4. Discussion

Fibrin is a natural biomaterial that supports cell adhesion, stimulates EC network formation and initiates healing and angiogenesis. Fibrin beads are a promising approach for robust, biochemically-active delivery of ECs for therapeutic applications. Previous attempts to generate fibrin beads used organic solvents which can drastically reduce cell viability. Here, we demonstrate a system with easily tunable parameters for fibrin bead synthesis, and culture techniques to support ECs for various tissue engineering applications including biosensor design.

In order to prepare fibrin microbeads there are a number of important considerations. Careful selection of alginate composition and concentration and thrombin concentration is required in order to produce spherical, solid fibrin beads. For example, utilizing low viscosity-high mannuronate (LVM) alginate, as well as reducing the LVG alginate concentration, resulted in non-spherical, teardrop-shaped scaffolds. Use of crude, uncharacterized alginate, as well as lower thrombin concentration, resulted in hollow fibrin beads.

Fibrin bead swelling ratios varied with initial fibrinogen concentration. As swell ratio correlates with gel stiffness, this suggests that a variety of scaffold mechanical properties are possible with the fibrin beads. This seems particularly useful as mechanical forces play great importance on cell signaling [38]. Particularly for ECs, literature suggests the spontaneous tube-like structure formation for bulk fibrin gels at fibrinogen concentrations less than 3 mg/mL [39]. Although we were not able to characterize the swelling ratios for beads made with fibrinogen concentrations under 5 mg/mL, we were able to form beads as low as 1 mg/mL. Further investigation on the role of scaffold stiffness is necessary to assess the potential of TPS culture of fibrin beads as a suitable approach for *in vitro* pre-vascularization strategies. We did not observe significant evidence of vascular structure formation, possibly due to the high concentration of fibrinogen used. Regardless, the delivery of endothelial cells in biomaterials has been shown to enhance neovascularization independent of pre-assembly [40]. In addition, we are currently investigating methods for inducing vessel assembly in the beads via variation in scaffold properties, bioreactor conditions and through the addition of support cells. 

The process of fibrin bead formation appears to result in cell viability consistent with other methods. While ECFC viability was 50%–60%, HUVEC data was on par with previous reports at greater than 70% [41]. This may result from the fact that ECFCs are particularly more sensitive to mechanical forces within the syringe. As such, a similarly low viability would be expected in other reports utilizing biomaterial encapsulated ECFCs. Another potential issue with ECFCs is variability across individual donors. Cell behavior varies with donor age, health and gender. A single donor was used for these studies, but, future work characterizing biomaterials-based ECFC approaches should address donor variability.

In long-term static culture, the majority of the ECFCs are dead by 7 days. Further, those that remain show higher levels of both moderate and severe DNA damage, compared to TPS-cultured ECFCs. This likely results from the significant diffusional barrier for nutrient transport in the large diameter 3D microbeads [42]. However, when cultured in a TPS, we are able to generate a convective flow around the beads, improving nutrients transport to cells in the center of the bead. At 7 days viability of the ECFCs in the TPS was high (94%) and cells were observed throughout the volume of the beads. The observed DNA damage of these cells is also minimal, similar to traditional 2D-cultured cells. By implementing a TPS, we are able to sustain ECFCs prior to implantation over the course of a week. Clinically, this is a major advantage to current implantation techniques, as it allows considerable flexibility in the timing of the surgical procedure for implantation of the material.

The TPS allows us to generate larger volumes of viable cells for treatment of clinically relevant volumes. In the context of sensors, this may enable development of multi-sensor systems that can be vascularized rapidly following implantation. This can be implemented by incorporating the beads into a coating on the sensor surface or implanting the beads near the sensor surface, as previously described [3]. A major advantage of fibrin is that it is already approved for clinical use, making this method suitable for clinical testing.

In such instances, manipulating the bead size is crucial for the survival of the scaffold. Specifically, it may be necessary to alter the microbead size in order to control physical properties that affect permeability and mass transfer within the microbeads [43,44]. Further, increasing the size of the beads may also be beneficial to ECs due to its influence on shear stress within the bioreactor [31], a factor that supports EC angiogenic signaling and proliferation [45]. In this study we were able to increase ECFC viability in fibrin beads through culture in the TPS system. While ECFCs have shown promise for vascularization of ischemic and engineered tissues, there has been little investigation of bioreactor culture systems to enhance their viability in culture. Dynamic culture in a biaxial bioreactor was shown to increase mineralization of ECs co-cultured with mesenchymal stem cells [46]. However, the impact on EC viability was not addressed.

## 5. Conclusions

In conclusion, we describe a method for enhancing ECFC viability in 3D fibrin beads using a tubular perfusion bioreactor for vascularization of biosensors. The fibrin beads provide a robust, tunable platform for delivery and culture of ECs. Further, a perfusion bioreactor can be utilized to culture these cells and prolong their viability pre-implantation. These scaffolds may hold promise as coating for biosensors to accelerate vascularization and improve long-term function.

## References

[1] Squires T.M., Messinger R.J., Manalis S.R. (2008). Making it stick: Convection, reaction and diffusion in surface-based biosensors. Nat. Biotechnol..

[2] Butt O.I., Carruth R., Kutala V.K., Kuppusamy P., Moldovan N.I. (2007). Stimulation of peri-implant vascularization with bone marrow-derived progenitor cells: Monitoring by *in vivo* EPR oximetry. Tissue Eng..

[3] Klueh U., Dorsky D.I., Kreutzer D.L. (2005). Enhancement of implantable glucose sensor function *in vivo* using gene transfer-induced neovascularization. Biomaterials.

[4] Kong H.J., Smith M.K., Mooney D.J. (2003). Designing alginate hydrogels to maintain viability of immobilized cells. Biomaterials.

[5] Nakatsu M.N., Sainson R.C.A., Aoto J.N., Taylor K.L., Aitkenhead M., Pérez-del-Pulgar S., Carpenter P.M., Hughes C.C.W. (2003). Angiogenic sprouting and capillary lumen formation modeled by human umbilical vein endothelial cells (HUVEC) in fibrin gels: The role of fibroblasts and Angiopoietin-1. Microvasc. Res..

[6] Nakatsu M.N., Davis J., Hughes C.C.W. (2007). Optimized fibrin gel bead assay for the study of angiogenesis. J. Vis. Exp..

[7] Barsotti M.C., Magera A., Armani C., Chiellini F., Felice F., Dinucci D., Piras A.M., Minnocci A., Solaro R., Soldani G. (2011). Fibrin acts as biomimetic niche inducing both differentiation and stem cell marker expression of early human endothelial progenitor cells. Cell Prolif..

[8] Mead L.E., Prater D., Yoder M.C., Ingram D.A. (2008). Isolation and characterization of endothelial progenitor cells from human blood. Curr. Protoc. Stem Cell Biol..

[9] Ingram D.A., Mead L.E., Tanaka H., Meade V., Fenoglio A., Mortell K., Pollok K., Ferkowicz M.J., Gilley D., Yoder M.C. (2004). Identification of a novel hierarchy of endothelial progenitor cells using human peripheral and umbilical cord blood. Blood.

[10] Hirschi K.K., Ingram D.A., Yoder M.C. (2008). Assessing identity, phenotype, and fate of endothelial progenitor cells. Arterioscler. Thromb. Vasc. Biol..

[11] Zigdon-Giladi H., Bick T., Lewinson D., Machtei E.E. (2013). Co-Transplantation of Endothelial Progenitor Cells and Mesenchymal Stem Cells Promote Neovascularization and Bone Regeneration. Clin. Implant Dent. Relat. Res..

[12] Chandrasekhar K.S., Zhou H., Zeng P., Alge D., Li W., Finney B.A., Yoder M.C., Li J. (2011). Blood vessel wall-derived endothelial colony-forming cells enhance fracture repair and bone regeneration. Calcif. Tissue Int..

[13] Chen B., Bo C.-J., Jia R.-P., Liu H., Wu R., Wu J., Ge Y.-Z., Teng G.-J. (2013). The renoprotective effect of bone marrow-derived endothelial progenitor cell transplantation on acute ischemia-reperfusion injury in rats. Transplant. Proc..

[14] Chung Y., Abou-Nassar K.E., Li Y., Filion L., Watpool I., McArdle T., McIntyre L., Ramsay T., Cheesman J., Touyz R. (2011). Vascular progenitor recruitment in critically ill patients with acute kidney injury. Clin. Investig. Med. Médecine Clin. Exp..

[15] Naruse K., Hamada Y., Nakashima E., Kato K., Mizubayashi R., Kamiya H., Yuzawa Y., Matsuo S., Murohara T., Matsubara T. (2005). Therapeutic neovascularization using cord blood-derived endothelial progenitor cells for diabetic neuropathy. Diabetes.

[16] Zhang Y., Li Y., Wang S., Han Z., Huang X., Li S., Chen F., Niu R., Dong J., Jiang R. (2013). Transplantation of expanded endothelial colony-forming cells improved outcomes of traumatic brain injury in a mouse model. J. Surg. Res..

[17] Moubarik C., Guillet B., Youssef B., Codaccioni J.-L., Piercecchi M.-D., Sabatier F., Lionel P., Dou L., Foucault-Bertaud A., Velly L. (2011). Transplanted late outgrowth endothelial progenitor cells as cell therapy product for stroke. Stem Cell Rev..

[18] Wei P., Milbauer L.C., Enenstein J., Nguyen J., Pan W., Hebbel R.P. (2011). Differential endothelial cell gene expression by African Americans versus Caucasian Americans: A possible contribution to health disparity in vascular disease and cancer. BMC Med..

[19] Allen P., Kang K.-T., Bischoff J. (2015). Rapid onset of perfused blood vessels after implantation of ECFCs and MPCs in collagen, PuraMatrix and fibrin provisional matrices. J. Tissue Eng. Regen. Med..

[20] Whittington C.F., Yoder M.C., Voytik-Harbin S.L. (2013). Collagen-polymer guidance of vessel network formation and stabilization by endothelial colony forming cells *in vitro*. Macromol. Biosci..

[21] Critser P.J., Voytik-Harbin S.L., Yoder M.C. (2011). Isolating and defining cells to engineer human blood vessels. Cell Prolif..

[22] Critser P.J., Kreger S.T., Voytik-Harbin S.L., Yoder M.C. (2010). Collagen matrix physical properties modulate endothelial colony forming cell-derived vessels *in vivo*. Microvasc. Res..

[23] Kang K.-T., Allen P., Bischoff J. (2011). Bioengineered human vascular networks transplanted into secondary mice reconnect with the host vasculature and re-establish perfusion. Blood.

[24] Gorodetsky R. (2008). The use of fibrin based matrices and fibrin microbeads (FMB) for cell based tissue regeneration. Expert Opin. Biol. Ther..

[25] Perka C., Arnold U., Spitzer R.S., Lindenhayn K. (2001). The use of fibrin beads for tissue engineering and subsequential transplantation. Tissue Eng..

[26] Bhakta G., Lee K.H., Magalhães R., Wen F., Gouk S.S., Hutmacher D.W., Kuleshova L.L. (2009). Cryopreservation of alginate-fibrin beads involving bone marrow derived mesenchymal stromal cells by vitrification. Biomaterials.

[27] Yeatts A.B., Fisher J.P. (2011). Tubular perfusion system for the long-term dynamic culture of human mesenchymal stem cells. Tissue Eng. Part C Methods.

[28] Yeatts A.B., Both S.K., Yang W., Alghamdi H.S., Yang F., Fisher J.P., Jansen J.A. (2014). *In vivo* bone regeneration using tubular perfusion system bioreactor cultured nanofibrous scaffolds. Tissue Eng. Part A.

[29] Prasain N., Meador J.L., Yoder M.C. (2012). Phenotypic and functional characterization of endothelial colony forming cells derived from human umbilical cord blood. J. Vis. Exp..

[30] Pisanti P., Yeatts A.B., Cardea S., Fisher J.P., Reverchon E. (2012). Tubular perfusion system culture of human mesenchymal stem cells on poly-L-lactic acid scaffolds produced using a supercritical carbon dioxide-assisted process. J. Biomed. Mater. Res. A.

[31] Yeatts A.B., Geibel E.M., Fears F.F., Fisher J.P. (2012). Human Mesenchymal Stem Cell Position within Scaffolds Influences Cell Fate During Dynamic Culture. Biotechnol. Bioeng..

[32] Yeatts A.B., Fisher J.P. (2011). Bone tissue engineering bioreactors: Dynamic culture and the influence of shear stress. Bone.

[33] Yeatts A.B., Gordon C.N., Fisher J.P. (2011). Formation of an aggregated alginate construct in a tubular perfusion system. Tissue Eng. Part C Methods.

[34] Yeatts A.B., Choquette D.T., Fisher J.P. (2013). Bioreactors to influence stem cell fate: Augmentation of mesenchymal stem cell signaling pathways via dynamic culture systems. Biochim. Biophys. Acta.

[35] Singh N.P., McCoy M.T., Tice R.R., Schneider E.L. (1988). A simple technique for quantitation of low levels of DNA damage in individual cells. Exp. Cell Res..

[36] Anderson D., Yu T.W., Phillips B.J., Schmezer P. (1994). The effect of various antioxidants and other modifying agents on oxygen-radical-generated DNA damage in human lymphocytes in the COMET assay. Mutat. Res..

[37] Zhao H., Ma L., Zhou J., Mao Z., Gao C., Shen J. (2008). Fabrication and physical and biological properties of fibrin gel derived from human plasma. Biomed. Mater. Bristol Engl..

[38] Sieminski A.L., Hebbel R.P., Gooch K.J. (2004). The relative magnitudes of endothelial force generation and matrix stiffness modulate capillary morphogenesis *in vitro*. Exp. Cell Res..

[39] Vailhé B., Ronot X., Tracqui P., Usson Y., Tranqui L. (1997). *In vitro* angiogenesis is modulated by the mechanical properties of fibrin gels and is related to alpha(v)beta3 integrin localization. In Vitro Cell. Dev. Biol. Anim..

[40] Nör J.E., Peters M.C., Christensen J.B., Sutorik M.M., Linn S., Khan M.K., Addison C.L., Mooney D.J., Polverini P.J. (2001). Engineering and characterization of functional human microvessels in immunodeficient mice. Lab. Investig. J. Tech. Methods Pathol..

[41] Aguado B.A., Mulyasasmita W., Su J., Lampe K.J., Heilshorn S.C. (2012). Improving viability of stem cells during syringe needle flow through the design of hydrogel cell carriers. Tissue Eng. Part A.

[42] Corstorphine L., Sefton M.V. (2011). Effectiveness factor and diffusion limitations in collagen gel modules containing HepG2 cells. J. Tissue Eng. Regen. Med..

[43] Mughal A., Chan H.K., Weaire D., Hutzler S. (2012). Dense packings of spheres in cylinders: Simulations. Phys. Rev. E Stat. Nonlin. Soft Matter Phys..

[44] Gautier A., Carpentier B., Dufresne M., Vu Dinh Q., Paullier P., Legallais C. (2011). Impact of alginate type and bead diameter on mass transfers and the metabolic activities of encapsulated C3A cells in bioartificial liver applications. Eur. Cell. Mater..

[45] Kaunas R., Kang H., Bayless K.J. (2011). Synergistic Regulation of Angiogenic Sprouting by Biochemical Factors and Wall Shear Stress. Cell. Mol. Bioeng..

[46] Liu Y., Teoh S.-H., Chong M.S.K., Yeow C.-H., Kamm R.D., Choolani M., Chan J.K.Y. (2013). Contrasting effects of vasculogenic induction upon biaxial bioreactor stimulation of mesenchymal stem cells and endothelial progenitor cells cocultures in three-dimensional scaffolds under *in vitro* and *in vivo* paradigms for vascularized bone tissue engineering. Tissue Eng. Part A.

